# Nd/TiO_2_ Anatase-Brookite Photocatalysts for Photocatalytic Decomposition of Methanol

**DOI:** 10.3389/fchem.2018.00044

**Published:** 2018-03-02

**Authors:** Kamila Kočí, Ivana Troppová, Martin Reli, Lenka Matějová, Miroslava Edelmannová, Helena Drobná, Lada Dubnová, Anna Rokicińska, Piotr Kuśtrowski, Libor Čapek

**Affiliations:** ^1^Institute of Environmental Technology, VŠB-Technical University of Ostrava, Ostrava, Czechia; ^2^Faculty of Chemical Technology, University of Pardubice, Pardubice, Czechia; ^3^Faculty of Chemistry, Jagiellonian University, Kraków, Poland

**Keywords:** neodymium, TiO_2_ anatase-brookite, CH_3_OH photocatalytic decomposition, hydrogen production, photocatalysis, electron-hole separation

## Abstract

Neodymium enriched TiO_2_ anatase-brookite powders were prepared by unconventional method via using pressurized hot fluids for TiO_2_ crystallization and purification. The photocatalysts were tested in the CH_3_OH photocatalytic decomposition and they were characterized with respect to the textural (nitrogen adsorption), structural (XRD, XPS, and Raman spectroscopies), chemical (XRF), and optical (DR UV-Vis spectroscopy) and photoelectrochemical measurement. All prepared materials were nanocrystalline, had biphasic (anatase- brookite) structure and relatively large specific surface area (125 m^2^.g^−1^). The research work indicates that the doping of neodymium on TiO_2_ photocatalysts significantly enhances the efficiency of photocatalytic reaction. The photocatalytic activity increased with increasing portion of hydroxyl oxygen to the total amount of oxygen species. It was ascertained that the optimal amount of 1 wt% Nd in TiO_2_ accomplished the increasing of hydrogen production by 70% in comparison with pure TiO_2_. The neodymium doped on the titanium dioxide act as sites with accumulation of electrons. The higher efficiency of photocatalytic process was achieved due to improved electron-hole separation on the modified TiO_2_ photocatalysts. This result was confirmed by electrochemical measurements, the most active photocatalysts proved the highest photocurrent responses.

## Introduction

Nowadays, a clean energy production from renewable sources is one of the most discussion topics. An excellent alternative to H_2_ production is the use of heterogeneous photocatalytic process. Since the pioneering work of Fujishima and Honda (1972) many research teams have focused to water splitting on semiconductor photocatalysts.

Due to the fact, that water splitting is difficult the reaction is often realized in the presence sacrificial reagent such as methanol. The addition of organic compounds such as alcohols to the water solution showed to be prosperous to overpass such limitations (Dozzi et al., 2017). In the presence of methanol (an electron donor), photogenerated the holes which are generated in the valence band can oxidize methanol in place of water. For this reason the reduction of water is decreasing by conduction band electrons. But it is necessary to the bottom of the conduction band to be situated above the water reduction potential (Maeda, 2011).

Amongst the possible photocatalysts for this application, titanium dioxide still remains the most investigated material, mainly because of its remarkable physical and chemical properties such as strong resistance to chemical and photocorrosion, low cost, and significantly low energy consumption (Ma et al., 2014; Schneider et al., 2014; Bai et al., 2015, 2016). However, the TiO_2_ application is limited due to its relatively high energy bandgap (3.2 eV) which required UV radiation and making it poor in the processes associated with solar photocatalytic applications (Xu and Song, 2016).

Rare earth elements doped or deposited on TiO_2_ are expected to show various effects on the TiO_2_ photocatalytic activity. The advantages of doping TiO_2_ with lanthanides can be namely (i) better TiO_2_ thermal stability, (ii) inhibition of TiO_2_ crystallite growth, (iii) limitation of the defect amount, and (iv) improvement of the photocatalytic activity (Meksi et al., 2016).

In the present article, the photocatalytic decomposition of methanol is studied in the presence of Nd/TiO_2_ anatase-brookite for the first time. These photocatalysts were prepared unconventionally via using flow processing by pressurized hot fluids for TiO_2_ crystallization and purification. This processing differs from the hydrothermal and solvothermal syntheses. While hydrothermal and solvothermal syntheses being carried out in a closed batch system, which is heated and pressurized, with liquid (aqueous or alcoholic) precursor solution inside, i.e., the precipitation and crystallization of nanostructured material occur simultaneously in one system which is heated and pressurized, the processing by pressurized hot fluids in our study is performed in a flow regime. The flow processing by pressurized hot fluids is performed and understood as a “post-treatment” of the gel precursors from sol-gel synthesis. This processing may be applied as a post-treatment of sol-gel derived precursors (formed gels) or precipitated amorphous solids as an alternative to standard calcination (thermal treatment). The flow arrangement of the pressurized hot fluid processing/crystallization offers following advantages: (i) faster heating, (ii) change of the chemical composition of the fluid/medium (e.g., to water, to water/methanol mixture, water/ethanol mixture) during the processing can affect the solubility of used organic precursors for-sol gel synthesis and, thus the purity and further crystallization of sol-gel derived nanomaterials. This processing results in nanocrystalline TiO_2_ of anatase-brookite crystal structure showing higher surface area then calcined analog of anatase crystal structure (Kocí et al., 2017). The aim of this study is to investigate the influence of the Nd dopant in TiO_2_ anatase-brookite on the H_2_ yields in photocatalytic decomposition of methanol.

## Materials and methods

### Nd/TiO_2_ preparation

Nd/TiO_2_ with various low neodymium (Nd) loadings (0.2–1.5 wt.%) and parent TiO_2_ were prepared by using a sol-gel process and a processing by pressurized hot fluids.

Chemicals for sol-gel synthesis: cyclohexane (HPLC grade), absolute ethanol (water content max. 0.2 vol.%), non-ionic surfactant Triton X 114 [(1,1,3,3-tetramethylbutyl)phenyl-polyethylene glycol, C_29_H_52_O_8.5_, Aldrich], titanium (IV) isopropoxide (99.999%, Aldrich), lanthanum(III) nitrate hexahydrate (Aldrich), neodymium(III) nitrate hexahydrate (Aldrich), and distilled water.

Neodymium doped TiO_2_ gels were synthesized via the sol-gel process controlled in the reverse micelles of non-ionic surfactant Triton X-114 in cyclohexane. Neodymium doped titania sols, resulting in gels after a gelation period, were prepared as follows: In the first step, the neodymium(III)-containing sol was prepared. The appropriate amount of neodymium(III) nitrate hexahydrate was dissolved in absolute ethanol (3 mL) under intense stirring. In the second step, cyclohexane was mixed with Triton X-114 and distilled water followed by the addition of neodymium(III)-containing sol. The sol was stirred for 20 min. In the final step, titanium (IV) isopropoxide was injected into the mixture. This neodymium doped titania micellar sol was stirred for next 20 min. Then the homogeneous yellow transparent sol was poured into Petri's dishes in a thin layer (~4 mm) and the dishes were left standing on air for gelation for 48 h. In general, the neodymium doped titania sols were prepared keeping the molar ratio of cyclohexane: Triton X-114: H_2_O: Ti(OCH(CH_3_)_2_)_4_+ Nd(NO_3_)_3_·6H_2_O at 11: 1: 1: 1 (Reli et al., 2015; Kocí et al., 2017). After the gelation period, the gels were ground to small pieces (~2 × 2 mm) and were processed by pressurized hot fluids.

Parent TiO_2_ was prepared by the same sol-gel process as mentioned above, but the preparation was simpler. In the first step, an appropriate amount of cyclohexane was mixed with Triton X-114 and distilled water and this sol was mixed for 20 min. After that titanium (IV) isopropoxide was injected into the mixture and titania micellar sol was stirred for next 20 min. The homogeneous transparent sol was poured into Petri's dishes in a thin layer (~4 mm) and the dishes were left standing on air for gelation for 48 h. In general, titania sol was prepared keeping the molar ratio of cyclohexane: Triton X-114: H_2_O: Ti(OCH(CH_3_)_2_)_4_ at 11: 1: 1: 1 (Matějová et al., 2010, 2012). After the gelation period, the gels were ground to small pieces (~2 × 2 mm) and were processed by pressurized hot fluids.

### Processing by pressurized hot fluids

Chemicals for processing: deionized water (electrical conductivity ~0.06–0.08 μS/cm) and methanol (HPLC grade).

The reasons of using both solvents (water and methanol) for gel processing were following: (i) water causes the crystallization of titania and dissolves a part of organic precursors, (ii) methanol dissolves the part of organic precursors being dissoluble in water. In final, excellently pure photocatalysts are prepared (Matějová et al., 2012).

Processing by pressurized hot water followed by processing by pressurized hot methanol was carried out in a laboratory-made unit equipped with a HPLC BETA10 Plus gradient pump (Ecom s.r.o., Czech Republic), a chromatographic oven operating in the temperature range of 25–400°C, capillary cooling and a restrictor operating at ambient temperature. A detailed description of the experimental setup is shown in Matejova et al. (2015) and Troppová et al. (2017). The gels, placed in 24-mL high-temperature stainless-steel cells, were processed in a flow regime at pressure 10 MPa and temperature 225°C, using the sequence of solvents 1.5 L deionized water −0.25 L methanol −0.1 L deionized water. The flow rates of solvents during the processing were kept at 3.8–4.2 mL/min. Produced powder photocatalysts were sieved to particle-size fraction <0.160 mm. This particle-size fraction was used for the characterizations as well as the photocatalytic tests.

### Characterization of Nd/TiO_2_ photocatalysts

Concentration (w/w) of Nd at TiO_2_ samples was analyzed with using Elva X energy-dispersive X-ray fluorescence spectrometer (Elvatech Ltd., Kiev, Ukraine) equipped with a Pd X-ray tube and thermoelectrically cooled Si-pin detector PF 550 (MOXTEC, USA). Power supply of X-ray tube was operated at 12 kV and 10 μA. The spectra at wavelength region of 0–10 keV were integrated for 120 s and Nd Lα line (5.2 KeV) was used as the analytical line. The sample chamber was flushed with He to suppress Ar interference. Powder samples were simply poured into plastic micro-vial covered by Mylar film. Every sample was analyzed at three replicates.

Nitrogen physisorption at 77 K was performed on a 3Flex automated volumetric apparatus (Micromeritics Instruments, USA) after degassing of materials at 150°C for more than 24 h under vacuum below 1 Torr. Degassing was applied to remove physisorbed water, but having no influence on the porous morphology of the developed materials. The specific surface area, S_BET_, was calculated according to the classical Brunauer–Emmett–Teller (BET) theory for the p/p0 range of 0.05–0.30 (Gregg and Sing, 1982). As the specific surface area, S_BET_, is not a proper parameter in the case of mesoporous solids containing micropores (Schneider, 1995), the mesopore surface area, S_meso_, and the micropore volume, V_micro_, were also evaluated based on the t-plot method (de Boer et al., 1966). The net pore volume, V_net_, was determined from the nitrogen adsorption isotherm at maximum p/p0 (~0.99). The pore-size distribution was evaluated from the adsorption branch of the nitrogen adsorption-desorption isotherm by the Barrett–Joyner–Halenda (BJH) method (Barrett et al., 1951) using the de Boer standard isotherm and assuming cylindrical pore geometry.

X-ray powder diffraction (XRD) patterns were obtained using a Rigaku SmartLab diffractometer (Rigaku, Japan) with detector D/teX Ultra 250. The source of X-ray irradiation was Co tube (CoKα, λ1 = 0.178892 nm, λ2 = 0.179278 nm) operated at 40 kV and 40 mA. Incident and diffracted beam optics were equipped with 5° Soller slits; incident slits were set up to irradiate area of the sample 10 × 10 mm (automatic divergence slits) constantly. Slits on the diffracted beam were set up to fixed value 8 and 14 mm. The powder materials were measured in the reflection mode (Bragg-Brentano geometry). The samples rotated (30 rpm) during the measurement to eliminate preferred orientation effect. The XRD patterns were collected in a 2θ range 5–90° with a step size of 0.01° and speed 0.5 deg.min^−1^. Measured XRD patterns were evaluated using PDXL 2 software (version 2.4.2.0) and compared with database PDF-2, release 2015. XRD patterns were analyzed using LeBail method (software PDXL2) to refine the lattice parameters of anatase. Background of the patterns was determined using the B-Spline function, peak shapes were modeled with a pseudo-Voigt function accounting for a peak asymmetry due to axial divergence. Crystallite size was calculated using Halder-Wagner method (software PDXL 2). Plotting β^2^/tan^2^θ against β/tan θ · sin θ based on the results makes it possible to obtain crystallite size from the gradient of the approximation line, where β is integral width of the sample diffraction peak and θ is diffraction peak position. Crystallite size of the anatase phase was calculated using reflections (101), (200), (105), (211), (116), and (220).

DRS spectra of the Nd/TiO_2_ materials were measured in quartz cuvettes by using a GBS CINTRA 303 spectrometer (GBC Scientific Equipment, Australia) equipped with integrating sphere. The spectra of catalysts were scanned in the wavelength range 190–900 nm, scan speed 100 nm.min^−1^, step size 1 nm and slit width of 2 nm. Reflectance was recalculated into the dependence of Kubelka–Munk function (Reli et al., 2015) based on the equation *F*(*R*_∞_) = (1-*R*_∞_)^2^/(2·*R*_∞_), where *R*_∞_ is the diffuse reflectance from a semi-infinite layer. This equation were transformed to the dependency (*F*(*R*_∞_)·*h*·ν)^1/2^ described as (α·*h*·ν)^1/2^ against photon energy for determination of band gap energy of indirect semiconductor.

Raman spectroscopy was measured using a Nicolet DXR SmartRaman spectrometer (Thermo Fisher Scientific, USA) equipped with 780 nm NIR excitation laser. The laser power on the sample was 1 mW, spectra were recorded by collecting of 200 scans and the spectrograph aperture was a 50 μm slit. Raman spectra were recorded in the 55–3,500 cm^−1^ wavenumber range.

XPS spectra were collected on a Prevac photoelectron spectrometer using Al Kα (E = 1486.6 eV) as a X-ray radiation source at a constant pass energy of 100 eV for survey and high resolution modes. A low energy electron flood gun (FS40A-PS) was used to compensate a surface charge. Powdered samples mounted on a sample holder were introduced by a load lock into an analytical chamber with a base pressure of 5 × 10^−9^ mbar. Binding energies of Ti 2p, O 1s, Nd 3d, Nd 4d, and C 1s photoelectron peaks were referenced to the C 1s core level (E_b_ = 285.0 eV). The fitting of high resolution spectra was provided through the Casa XPS software.

Photoelectrochemical measurements were conducted using classical three electrode system (Instytut Fotonowy, Poland), where Pt wire and saturated Ag/AgCl were used as counter and reference electrodes, respectively. The working electrode was prepared according to the following procedure: Small amount of photocatalyst was placed into a mortar and ground to a fine powder. Afterwards, two drops of demineralized water were added and suspension was created. Finally, the suspension was deposited on the conductive side of the ITO foil using the pestle. The suspension was deposited in a way it created uniform layer. The ITO foil with the suspension was into a hot air stream (hair drier) until the suspension dried out. The active area of electrode was 41 mm^2^. The 0.1M KNO_3_ solution was used as an electrolyte. Photoelectric spectrometer was coupled with potentiostat and the 150 W Xe lamp was used as irradiation source. In order to obtain better resolution around 300 nm grating was used. Photocurrent responses were measured in the wavelength range of 240–500 nm (10 nm step) and the potential range of −0.2 to 1.0 V (0.1 V step). The free oxygen environment was reached by purging the electrolyte in measuring cell by argon for 15 min before the measurement itself and during the whole measurement as well. Photoelectrochemical measurements were conducted in order to compare the amount of generated charge carriers for each photocatalyst.

### Photocatalytic experiments

The photocatalytic decomposition of methanol was performed in a homemade stirred batch photoreactor illuminated by UV 8 W Hg lamp (365 nm) under ambient temperature and pressure. The photocatalysts powder (concentration 1g.L^−1^) was suspended in methanol solution. Firstly, argon was purged through the suspension with a constant flow to remove air and then the reactor was sealed. The reaction products were analyzed by GC/BID. The photocatalytic reaction was done in time intervals of 0–4 h. The details of the photocatalytic decomposition of methanol were depicted in our previous publication (Kocí et al., 2017).

## Results

### Physico-chemical properties of investigated photocatalysts

**Figure 1:**
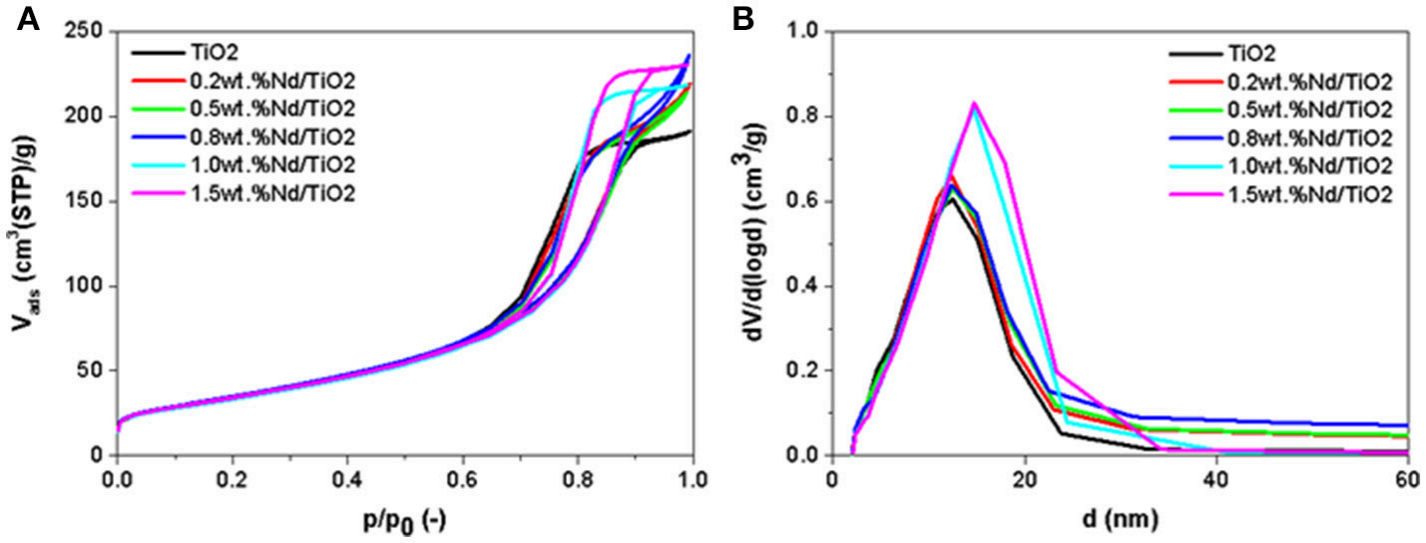
**(A)** Measured nitrogen adsorption-desorption isotherms and **(B)** evaluated pore-size distributions of investigated photocatalysts.

Measured nitrogen physisorption isotherms, evaluated pore-size distributions and determined textural properties of investigated photocatalysts including parent TiO_2_ are shown in Figures 1A,B and Table 1. It is evidenced (Figures 1A,B) that all the photocatalysts are mesoporous solids with negligible comparable amount of micropores (~31 ${\text{mm}}_{\text{liq}}^{3}$.g^−1^). Nd doped TiO_2_-based photocatalysts show also similar volume and size-distribution of pores (Figures 1A,B and Table 1).

**Table 1:** Chemical composition, textural, and optical properties of investigated photocatalysts.

**Photocatalyst**	**XRF**	**Physisorption**		**DRS UV-vis**
	**The real content of Nd (wt.%)**	**S*_*BET*_* (m^2^.g^−1^)**	**V*_*net*_* (${\text{mm}}_{\text{liq}}^{3}$.g^−1^)**	**Indirect band gap (eV)**
TiO_2_	–	125	296	3.24
0.2 wt.% Nd/TiO_2_	0.20	123	339	3.26
0.5 wt.% Nd/TiO_2_	0.52	121	331	3.26
0.8 wt.% Nd/TiO_2_	0.82	125	363	3.26
1.0 wt.% Nd/TiO_2_	0.95	120	338	3.19
1.5 wt.% Nd/TiO_2_	1.47	123	357	3.18

**Figure 2:**
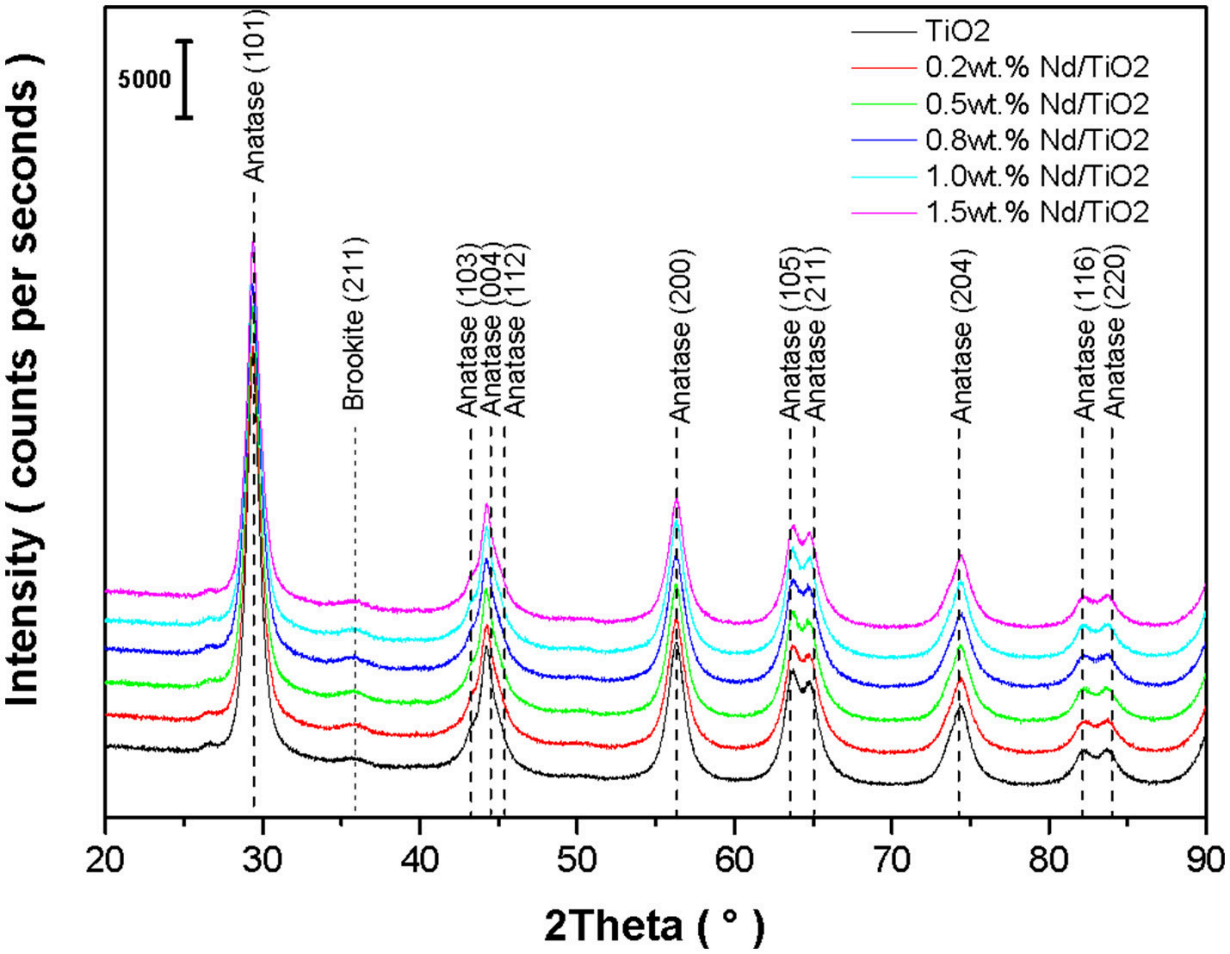
XRD patterns of investigated Nd/TiO_2_ photocatalysts and parent TiO_2_.

XRD patterns of pure TiO_2_ and Nd^3+^ ions doped TiO_2_ are shown in Figure 2. Presented diffraction lines correspond to the tetragonal TiO_2_ anatase (PDF-2 card No. 00-021-1272) and to the orthorhombic TiO_2_ brookite (PDF-2 card No. 01-071-4943). Brookite was detected in parent TiO_2_ as well as in all Nd/TiO_2_, thus the presence of brookite arises from the preparation process, namely the pressurized hot fluids processing, and it is not affected by Nd^3+^ ions doping. All the investigated photocatalysts are of anatase-brookite crystal structure. The effect of the preparation process on the crystallization of TiO_2_ was also observed in Gotić et al. (1996) and Musić et al. (1997). Any additional polymorphic forms of TiO_2_ (e.g., rutile) or Nd related phases (e.g., Nd_2_O_3_, Nd titanates) were not detected. Refined TiO_2_ anatase crystallite size, anatase lattice parameters, and cell volumes are summarized in Table 2. It is evident that the anatase lattice parameters and cell volume did not change with Nd addition. The anatase crystallites changed in the range of 8.5–10.2 nm. In the case of Nd^3+^ doped TiO_2_ photocatalysts, Nd^3+^ ions could go to the interstitial positions as a consequence of ionic radii; Nd^3+^ is 1.123 Å large and Ti^4+^ is about 0.745 Å. From this reason any substitution of Nd^3+^ ion for Ti^4+^ ion in the TiO_2_ lattice would introduce a distortion (Jalajakumari et al., 1999).

**Table 2:** Structural and microstructural properties of investigated photocatalysts.

**Photocatalyst**	**Anatase crystallite size (nm)**	Amount of brookite^*^	**Lattice parameters and cell volume of anatase**		
		**(wt. %)**	***a* (Å)**	***c* (Å)**	**V*_*cell*_* (Å^3^)**
TiO_2_	10.2	6.6	0.3791	0.9487	13.633
0.2 wt.% Nd/TiO_2_	8.8	11.9	0.3791	0.9485	13.628
0.5 wt.% Nd/TiO_2_	9.2	11.6	0.3792	0.9498	13.653
0.8 wt.% Nd/TiO_2_	8.5	13.1	0.3796	0.9493	13.676
1.0 wt.% Nd/TiO_2_	9.5	12.8	0.3790	0.9483	13.618
1.5 wt.% Nd/TiO_2_	9.7	11.5	0.3793	0.9477	13.632

* *Determined from Raman spectra*.

**Figure 3:**
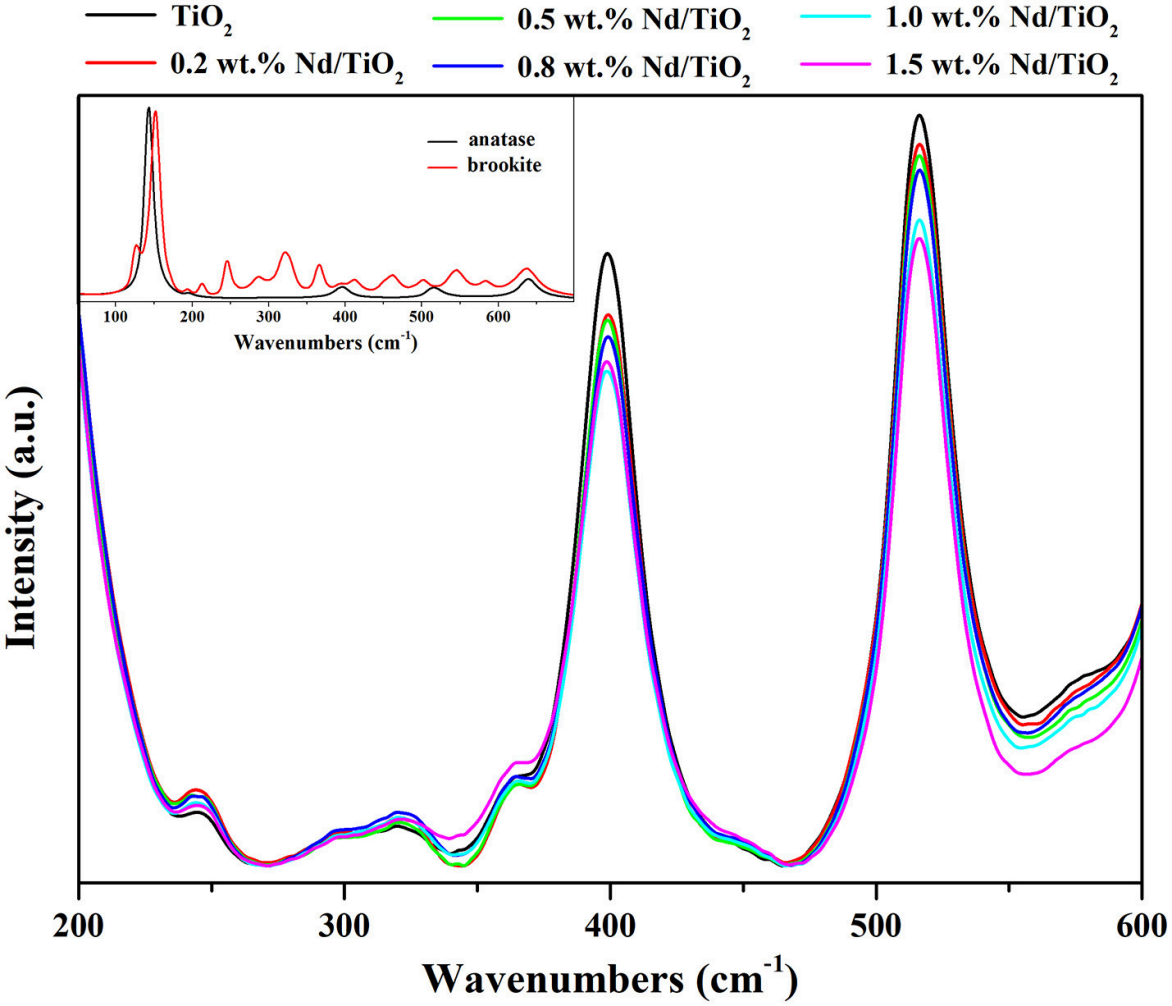
Raman spectra of Nd/TiO_2_ photocatalysts (main graph) and of pure TiO_2_ anatase and brookite phases (inlet graph).

Phase composition of Nd/TiO_2_ photocatalysts was determined by Raman spectroscopy (Figure 3). The Raman spectra of pure anatase and brookite forms of TiO_2_ are shown at inlet graph of Figure 3. Anatase form of TiO_2_ exhibits the bands at 143, 195, 396, 516, and 640 cm^−1^. In contrast to anatase form of TiO_2_, Raman spectrum of brookite phase of TiO_2_ contains more than 15 bands in the range from 50 to 700 cm^−1^. The phase composition was determined from the most intensive independent bands at 245 cm^−1^ for brookite phase and at 516 cm^−1^ for anatase phase. The ratios of these two bands indicate the brookite weight content (Table 2). While the lowest brookite phase content exhibits pure TiO_2_, the highest one contains Nd/TiO_2_ photocatalyst with 0.8 wt.% of Nd.

**Figure 4:**
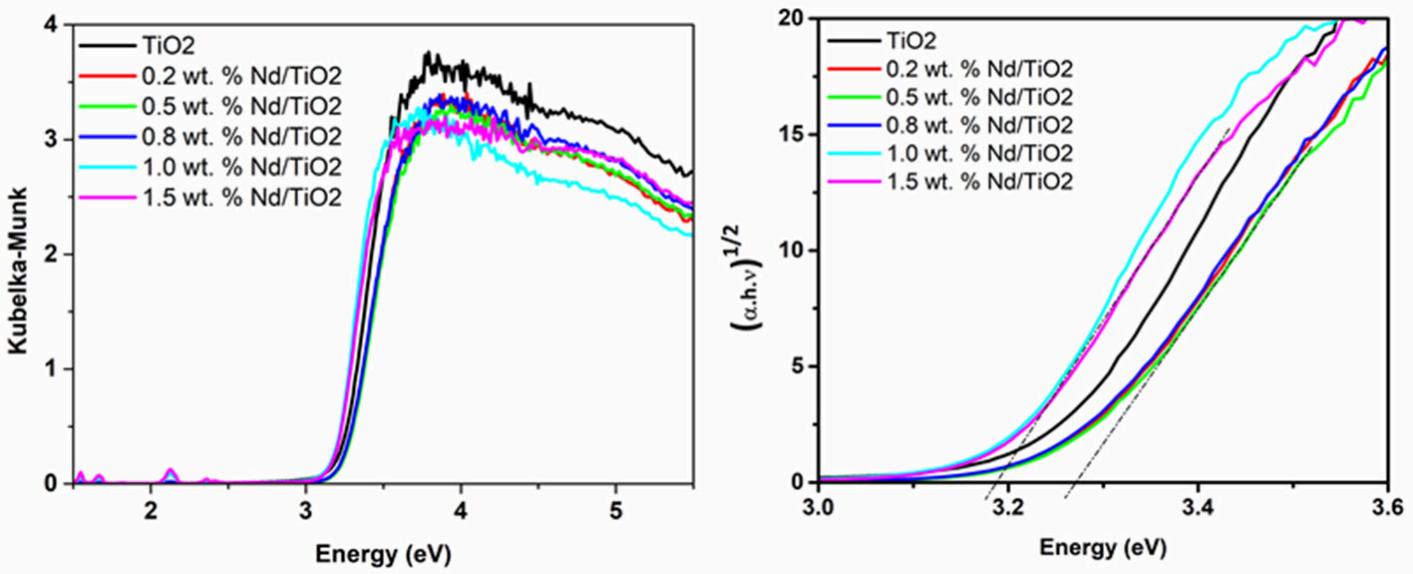
UV-vis DRS spectra of pure TiO_2_ and Nd/TiO_2_ photocatalyst.

Figure 4 shows diffuse reflectance spectra that were recalculated to the dependencies (α·*h*·ν)^1/2^ against energy in order to obtain the values of the indirect band gap energies that value was approximately the same for all studied Nd/TiO_2_ materials (Table 1).

**Figure 5:**
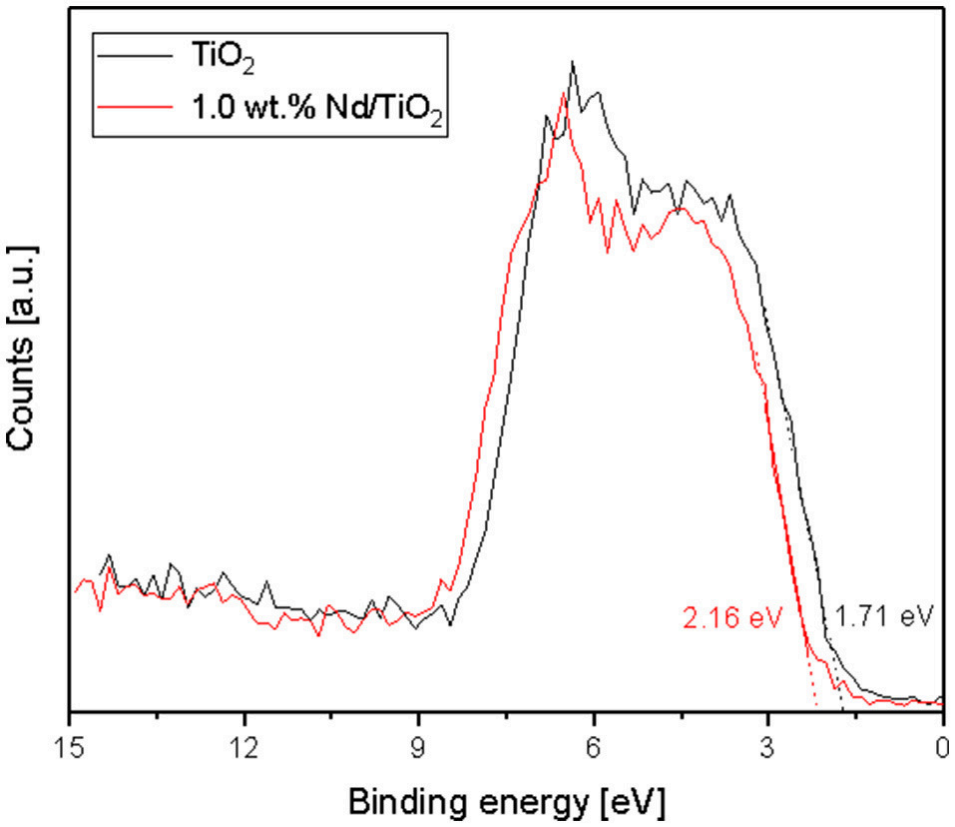
XPS valence-band spectra of bare TiO_2_ and 1.0 wt.% Nd/TiO_2_.

Four various elements were identified on the photocatalyst surface in the survey XPS spectra—Ti, O, Nd, and contaminating C. Unfortunately, the determination of neodymium content was practically impossible due to overlapping of Nd 3d peaks with O KLL Auger lines and very low intensity of Nd 4d peaks. The surface concentration of the other main components is demonstrated in Table 3. The Nd-free TiO_2_ shows two photoelectron peaks at 458.1 and 463.8 eV, which correspond to Ti 2p_3/2_ and Ti 2p_1/2_ levels and confirm the existence Ti^4+^ exclusively (Reli et al., 2017). A shift of these peaks to higher binding energies (about 0.2–0.3 eV) is observed after the modification of TiO_2_ with Nd. This finding confirms the strong interaction of introduced Nd ions with the titania structure. In the O 1s spectra, two forms of surface oxygen can be distinguished. The peak at 529.3–529.7 eV is related to the photoemission from lattice O^2−^, whereas another peak at 530.6–531.4 eV corresponds to the presence of hydroxyls (Reli et al., 2016). Changes in the distribution of both oxygen components are found after the doping of TiO_2_ with Nd. After the introduction of the lowest amount of Nd—only 7.3% of surface O forms hydroxyls. For the photocatalysts with higher Nd loadings the contribution of OH^−^ becomes much more akin to that of undoped TiO_2_. Furthermore, the valence band maximum (VBM) energy levels were analyzed based on the collected XPS spectra. The VBM values were calculated as the distance between 0 eV (the Fermi energy level) and the X intercept obtained by extrapolation of the descending part of the signal, as shown in Figure 5. The bare TiO_2_ exhibits the VBM of 1.71 eV, whereas this material modified with 1 wt.% of Nd −2.16 eV. So clear shift in the VBM values can be explained by various phase compositions of both samples. As is presented in Table 2, the content of brookite increases from 6.6 (pure titania) to 12.8 wt.% (titania containing Nd). The previous studies for different TiO_2_ polymorphs revealed that brookite has a conduction band minima higher in energy than anatase or rutile (Buckeridge et al., 2015).

**Table 3:** Surface composition of Nd-doped TiO_2_ photocatalysts determined by XPS.

**Photocatalyst**	**Ti^4+^ in TiO_2_ (at.%)**	**Oxygen**		
		**Lattice O^2−^ (at.%)**	**OH^−^ (at.%)**	**Portion of OH^−^ oxygen to total oxygen (at.%)**
TiO_2_	26.7	54.9	8.3	13.1
0.2 wt.% Nd/TiO_2_	28.2	60.8	4.8	7.3
0.5 wt.% Nd/TiO_2_	28.6	58.7	6.8	10.4
0.8 wt.% Nd/TiO_2_	26.1	55.5	6.4	10.3
1.0 wt.% Nd/TiO_2_	26.2	55.7	6.9	11.0
1.5 wt.% Nd/TiO_2_	26.0	55.2	6.6	10.7

**Figure 6:**
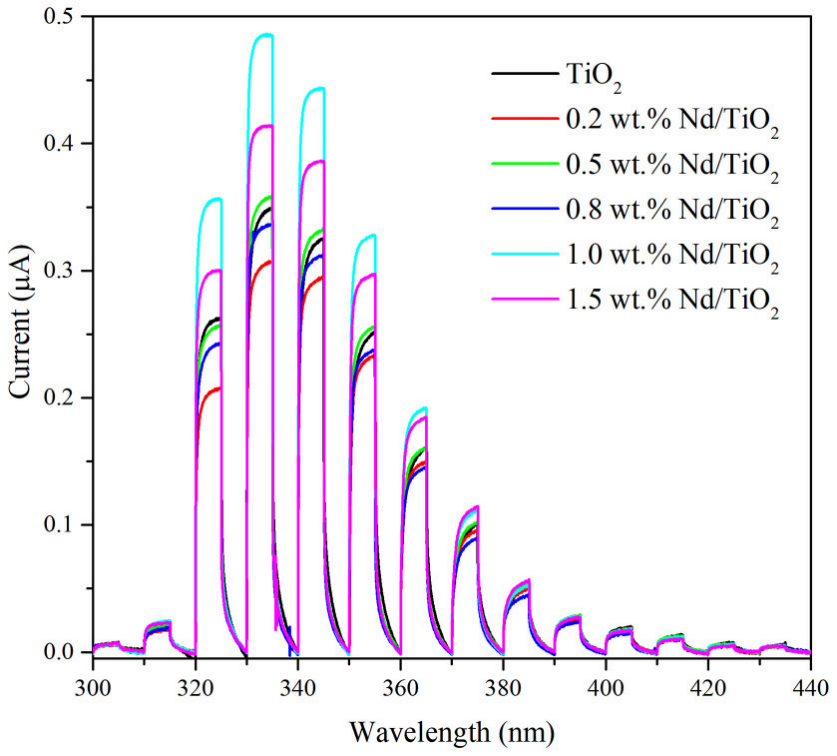
Current generation in the presence of Nd/TiO_2_ and parent TiO_2_ photocatalysts at 1 V vs. Ag/AgCl.

The photoelectrochemical properties of the photocatalyst are essential in the photocatalysis. The photocurrent measurements are useful to predict the photocatalytic activity of the photocatalyst or to discuss if the photocatalyst is capable of generation of electron-hole pairs under specific wavelength irradiation. The dependence of the generated current on wavelength in the presence of each tested photocatalyst is shown in Figure 6. The depicted current dependencies were measured in the presence of maximum applied potential of 1,000 mV. The potential was applied in order to separate the generated electrons and holes. The measurements confirmed that each of the prepared photocatalysts is generating rapid increase of current under irradiation in the range of wavelength of 320–380 nm. Unfortunately, the current response under 320 nm is not observed due to technical limitations of the device.

### The photocatalytic decomposition of CH_3_OH

**Figure 7:**
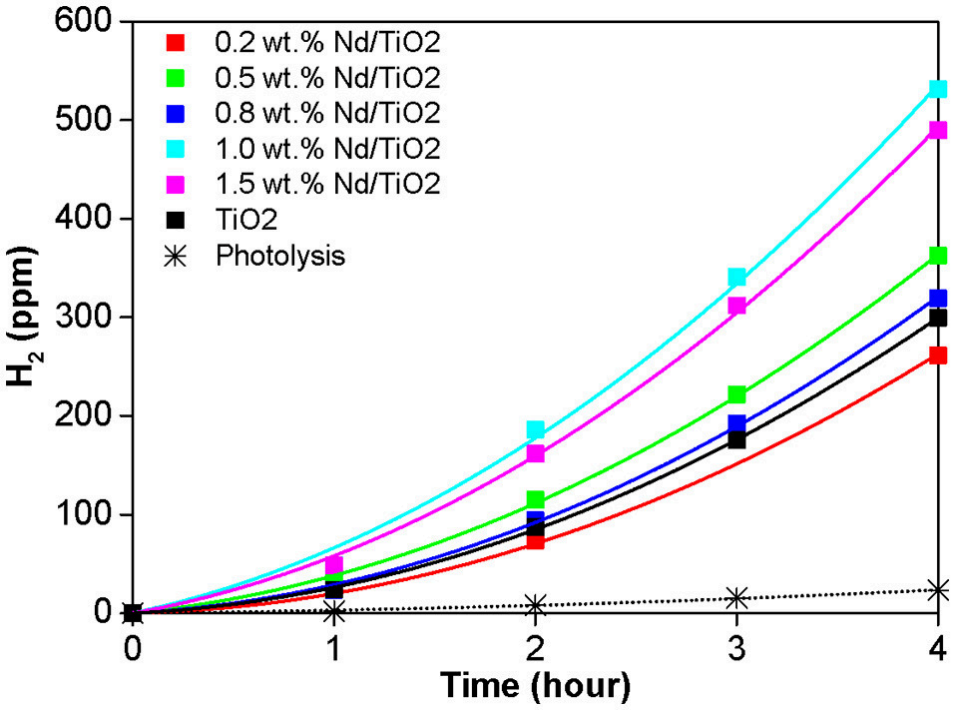
Generation of hydrogen in the photocatalytic oxidation of methanol in the presence of Nd/TiO_2_ and parent TiO_2_ photocatalysts.

The photocatalytic evolution of hydrogen during UVA illumination of the pure TiO_2_, and Nd/TiO_2_ photocatalysts in methanol decomposition is shown in Figure 7. All studied photocatalysts possessed significantly higher amount of the formed H_2_ in comparison with photolysis. All doped photocatalysts shown higher production of hydrogen than pure TiO_2_ with exception of 0.2 wt.% Nd. The Nd photocatalysts performance is decreasing in ranking: 1 wt.% Nd/TiO_2_ > 1.5 wt.% Nd/TiO_2_ > 0.5 wt.% Nd/TiO_2_ > 0.8 wt.% Nd/TiO_2_ > TiO_2_ > 0.2 wt.% Nd/TiO_2_. These trends indicate the existence of optimum Nd loading in TiO_2_ anatase-brookite photocatalyst working under UVA light. The 1 wt.% Nd/TiO_2_ had 70% higher activity as compared to undoped one.

## Discussion

The Nd/TiO_2_ photocatalyst absorbed photons from UVA irradiation and generated electron-hole pairs. However, the recombination of h^+^/e^−^ is one the most substantial elements, which can decrease the photoactivity (Du et al., 2013). The higher photocatalytic activity of Nd/TiO_2_, can be because of a combination of the following factors: (i) the trapping of electrons and holes to lead decreasing recombination of h^+^/e^−^ pairs during photocatalytic reaction, (ii) portion of lattice and surface O species to total oxygen, and (iii) the optimal crystallite size.

**Figure 8:**
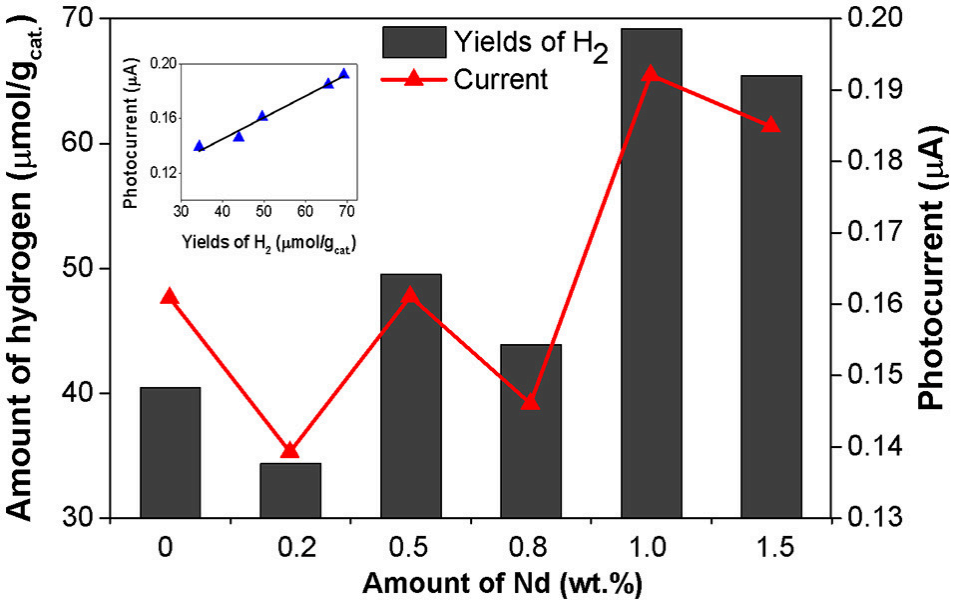
Correlation between the photocatalytic activity in the CH_3_OH photocatalytic decomposition in the presence of Nd/TiO_2_ photocatalysts and current generation. Current responses were obtained at 360 nm under external potential of 1.0 V.

Figure 7 shows the correlation between the photocurrent response and the amount of formed hydrogen. This result confirms the ability of the Nd species in separation of photogenerated electron–hole pairs (Nie et al., 2013; Siah et al., 2017). The 1.0 wt.% Nd/TiO_2_ photocatalyst had both the highest current response and the photocatalytic activity as well (Figure 8). This is clearly pointing toward the optimal neodymium loading, which is around 1 wt.%. Altogether, the trend of current responses of Nd/TiO_2_ photocatalysts matches their photocatalytic activity trend (Figure 8 inset). This result is suggesting there is a better separation of charge carriers. Low current means lower amount of charge carriers generated after irradiation but moderate photocatalytic activity is pointing toward their better utilization.

**Figure 9:**
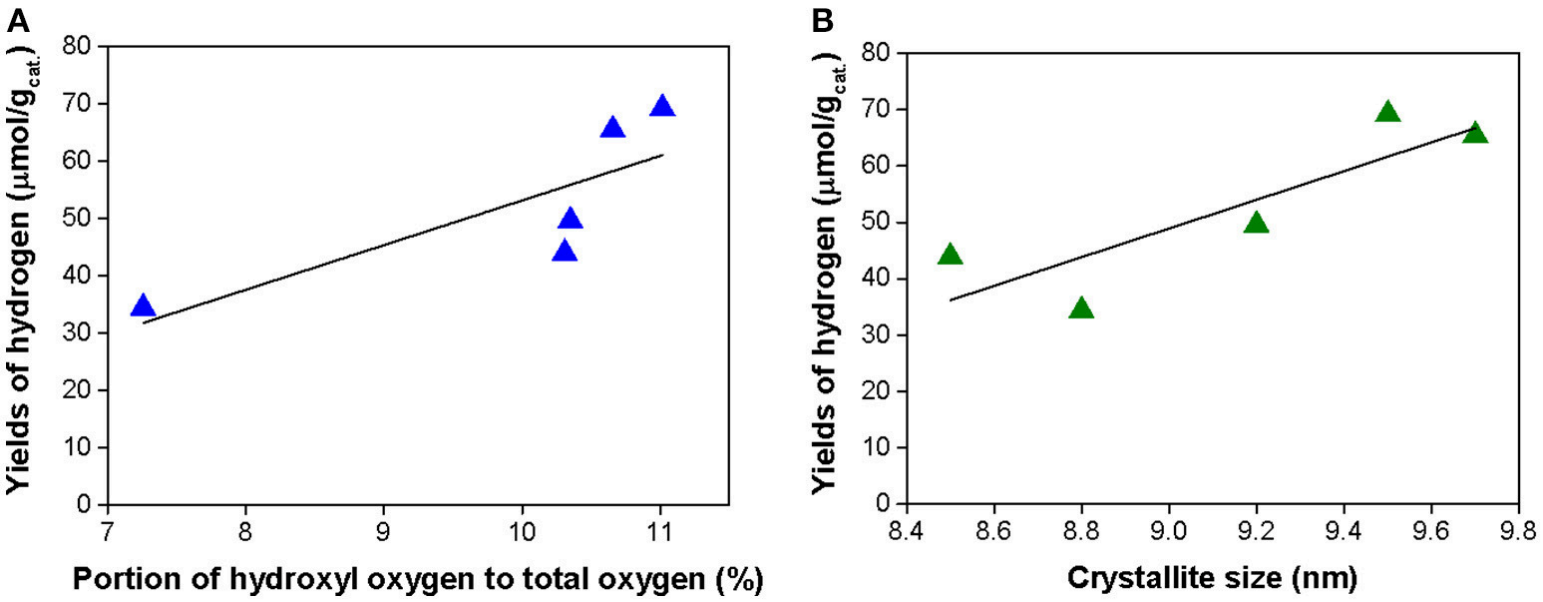
Correlation between the yield of hydrogen in the CH_3_OH photocatalytic oxidation and content of lattice oxygen to sum of oxygen **(A)** and crystallite size **(B)**.

It is evident from nitrogen physisorption, XRD and DR UV-Vis results that the Nd dopation did not cause any significant differences in the surface area and band gap energy of Nd/TiO_2_ photocatalysts. Contrary to that some differences in the surface properties were recognized, namely in the surface hydroxylation of photocatalysts. Nd/TiO_2_ photocatalysts differ in the proportional representation of lattice oxygen and oxygen comprised in surface OH^−^ (hydroxyl) groups. The hydrogen yields increased with the increasing amount of hydroxyls species obtained from XPS (Figure 9A). The highest yield of hydrogen was achieved for 1 wt.% Nd/TiO_2_ photocatalyst with the highest portion of hydroxyl O species to the total amount of oxygen (11%). The hydroxyl free radicals can be formed by reaction of the photogenerated holes with surface hydroxyl groups. Hydroxyl free radicals are a strong oxidizing agent and significantly support the separation of the h^+^/e^−^ pairs (Lu et al., 2011). Similarly, they can serve as adsorption sites for methanol or water. Therefore, the accrual of surface hydroxyl groups can make easier to the improvement of photoactivity.

Finally, the crystallite size of anatase is also one of the parameter in the photocatalytic reaction. The very small particles exhibit less photocatalytic activity. Correlation of crystallite size and photoactivity of investigated Nd/TiO_2_ is shown in Figure 9B. This phenomenon can be caused by two factors: (i) reduction of the crystallite size increased recombination centers and consequently it led to decreased activity (Grela and Colussi, 1996; Zhang et al., 1998; Lin et al., 2006; Kočí et al., 2009) and (ii) due to the flocculation of very small particles, the number of active sites can reduce (Maira et al., 2000; Lin et al., 2006). The 0.2 wt.% Nd/TiO_2_ and 0.8 wt.% Nd/TiO_2_ proved the smallest particle size (8.8 and 8.5 nm, respectively) and the smallest photoactivity, simultaneously. Beside the crystallite size also the portion of hydroxyl oxygen to total oxygen influence the final photocatalytic activity of the photocatalyst. These two factors are the reason why 0.2 wt.% Nd/TiO_2_ showed lower photocatalytic activity than pure TiO_2_. The 0.2 wt.% Nd/TiO_2_ has not only small crystallite size but also the lowest portion of hydroxyl oxygen to total oxygen.

It was found that an appropriate neodymium amount had a beneficial effect on the efficiency of methanol decomposition. In case of 1 wt.% Nd/TiO_2_ the decomposition of methanol was the most dynamic and this photocatalyst had the highest photocatalytic activity. Slightly worse results were obtained for the 1.5 wt.% Nd/TiO_2_. This means that there is an optimum neodymium amount. Khalid et al. (2013) studied methyl orange degradation in the presence Nd/TiO_2_ photocatalysts. In their studies, 1 at. % of Nd was an optimum value for the photocatalysis, and the efficiency of methyl orange decomposition was 20% better than for pure titania. Bokare et al. (2013) also got at the similar result. He dealt with the investigation of antibacterial activities Nd doped TiO_2_ photocatalyst. In the case of their TiO_2_:Nd nanopowders, the optimal value of Nd was 1 at. %, which resulted in 30% higher activity in comparison with undoped one. Wojcieszak et al. (2014) reporter for methyl orange degradation the optimal amount of Nd doped to be 3 at. %. The same optimal amount of Nd was found also by Rengaraj et al. (2007) during photoreduction of Cr(VI).

It also should be mentioned that previously we focused on lanthanoids doped TiO_2_ photocatalysts (La/TiO_2_) prepared by sol-gel method followed by the calcination of material at 500°C. In that case, La/TiO_2_ with 0.2 wt. % La showed higher amount of hydrogen formed in the methanol decomposition than La/TiO_2_ with 1.0 wt. % La (Kocí et al., 2017), but it should be stressed that the amount of formed hydrogen is almost 5 times higher for Nd/TiO_2_ photocatalysts prepared by unconventional method via using pressurized hot fluids for TiO_2_ crystallization and purification (Figure 7) in contrast to La/TiO_2_ photocatalysts prepared by sol-gel method followed by the calcination of material at 500°C (Kocí et al., 2017). However, while La/TiO_2_ photocatalysts prepared by sol-gel method followed by the calcination represented pure anatase form, Nd/TiO_2_ photocatalysts prepared by unconventional method via using pressurized hot fluids for TiO_2_ crystallization and purification represent anatase-brookite phase. It could be suggested that the biphasic photocatalysts show higher activity then monophasic photocatalysts. Biphasic photocatalysts allowed more effective separation of generated electron-hole pair thereby increased efficiency of photocatalytic reaction. These interesting results prove that unconventionally prepared Nd/TiO_2_ anatase-brookite based photocatalysts could greatly enhance the methanol photocatalytic decomposition, enhancing hydrogen production.

The betterment of the photocatalytic activity of TiO_2_ for the methanol decomposition due to the dopation of Nd can be explained by the following mechanisms: Nd on titanium dioxide behave as electron traps, increasing the e^−^/h^+^ separation and the ensuing transmittal of the trapped e^−^ to the adsorbed H^+^ which serves as an electron acceptor. This can be described by the following mechanism. The photocatalyst absorbed photons from UVA irradiation and generated of electrons and holes (Equation 1). The presence of Nd can easy the e^−^/h^+^ separation process by attracting e^−^ (Equation 2; Rengaraj et al., 2007). This reaction allows the holes h^+^ to react with the adsorbed CH_3_OH and H_2_O to form H^+^ (Equations 3 and 4). In the same way, the e^−^ can react with H^+^ to H_2_ (Equation 5). The presence of Nd on the titanium dioxide favors the transfer of photogenerated e^−^ to Nd, so improving the separation of charge carriers. Thereafter, the e^−^ migrate from Nd to the H^+^ ion and formed H_2_ molecules.

(1) $$\begin{array}{r} TiO_{2}+hv\to e^{-}+h^{+} \end{array}$$

(2) $$\begin{array}{r} Nd+e^{-}\to e_{Nd^{-}} \end{array}$$

(3) $$\begin{array}{r} CH_{3}OH+2h^{+}\to HCHO+2H^{+} \end{array}$$

(4) $$\begin{array}{r} 2H_{2}O+4h^{+}\to 4H^{+}+O_{2} \end{array}$$

(5) $$\begin{array}{r} 6H^{+}+6e^{-}\to 3H_{2} \end{array}$$

## Conclusion

The photocatalytic properties of the investigated Nd/TiO_2_ photocatalysts were evaluated by photocatalytic decomposition of CH_3_OH. The all as-prepared materials had anatase- brookite nanocrystalline structure. The specific surface area was relatively large (125 m^2^.g^−1^) and it was independent on the amount of Nd. On the other hand, Nd doping caused differences in surface properties namely in the surface hydroxylation of photocatalysts. Thanks to neodymium doping, the efficiency of photocatalytic decomposition of methanol was substantially increased in comparison with undoped TiO_2_ with exception of Nd/TiO_2_ with 0.2 wt.% Nd. The optimum amount of neodymium was established as 1 wt. % Nd. This photocatalyst had the highest portion of hydroxyl O species to the total amount of oxygen, which can facilitate to the improvement of photoactivity. The neodymium which was doped on the TiO_2_, act as sites with accumulation of electrons. The better separation of charge carriers on the modified TiO_2_ permits direction of the electrons and holes into the desirable oxidation and reduction reactions that is more efficient than the recombination reactions. This conclusion was also confirmed by electrochemical measurements.

## Author contributions

KK and LČ: Analysis of the correlation between the structural, textural, optical, electrochemical properties and the photocatalytic activity, preparation of publication; IT: Preparation of photocatalysts and its characterization by N_2_ adsorption; MR: Characterization of photocatalysts by photoelectrochemical measurement, analysis of the correlation between the structural, textural, optical, electrochemical properties and the photocatalytic activity; LM: Evaluation of N_2_ physisorption and correlation of the data with other characterization techniques; ME: photocatalytic tests; HD: Characterization of photocatalysts by Raman spectroscopy; LD: Preparation of photocatalysts and its characterization by using DRS; AR: Characterization of photocatalysts by using XPS; PK: Evaluation of XPS data and correlation of the results with photocatalytic data.

### Conflict of interest statement

The authors declare that the research was conducted in the absence of any commercial or financial relationships that could be construed as a potential conflict of interest.

## References

[B1] Bai S. Jiang J. Zhang Q. Xiong Y. (2015). Steering charge kinetics in photocatalysis: intersection of materials syntheses, characterization techniques and theoretical simulations. Chem. Soc. Rev. 44, 2893–2939. 10.1039/C5CS00064E 25904385

[B2] Bai S. Yin W. Wang L. Li Z. Xiong Y. (2016). Surface and interface design in cocatalysts for photocatalytic water splitting and CO_2_ reduction. RSC Adv. 6, 57446–57463. 10.1039/C6RA10539D

[B3] Barrett E. P. Joyner L. G. Halenda P. P. (1951). The determination of pore volume and area distributions in porous substances .1. Computations from nitrogen isotherms. J. Am. Chem. Soc. 73, 373–380.

[B4] Bokare A. Sanap A. Pai M. Sabharwal S. Athawale A. A. (2013). Antibacterial activities of Nd doped and Ag coated TiO_2_ nanoparticles under solar light irradiation. Colloids Surf. B Biointerfaces 102, 273–280. 10.1016/j.colsurfb.2012.08.030 23010118

[B5] Buckeridge J. Butler K. T. Catlow C. R. A. Logsdail A. J. Scanlon D. O. Shevlin S. A. (2015). Polymorph engineering of TiO_2_: demonstrating how absolute reference potentials are determined by local coordination. Chem. Mater. 27, 3844–3851. 10.1021/acs.chemmater.5b00230

[B6] de Boer J. H. Lippens B. C. Linsen B. G. Broekhof J. C. P. van den Heuvel A. Osinga T. J. (1966). T-curve of multimolecular N_2_-adsorption. J. Colloid Interface Sci. 21, 405–414.

[B7] Dozzi M. V. Chiarello G. L. Pedroni M. Livraghi S. Giamello E. Selli E. (2017). High photocatalytic hydrogen production on Cu(II) pre-grafted Pt/TiO_2_. Appl. Catal. B 209, 417–428. 10.1016/j.apcatb.2017.03.007

[B8] Du J. Chen H. Yang H. Sang R. Qian Y. Li Y. (2013). A facile sol–gel method for synthesis of porous Nd-doped TiO_2_ monolith with enhanced photocatalytic activity under UV–Vis irradiation. Microporous Mesoporous Mater. 182, 87–94. 10.1016/j.micromeso.2013.08.023

[B9] Fujishima A. Honda K. (1972). Electrochemical photolysis of water at a semiconductor electrode. Nature 238, 37–38. 10.1038/238037a0 12635268

[B10] Gotić M. Ivanda M. Sekulić A. Musić S. Popović S. Turković A. (1996). Microstructure of nanosized TiO_2_ obtained by sol-gel synthesis. Mater. Lett. 28, 225–229.

[B11] Gregg S. J. Sing K. S. W. (1982). Adsorption, Surface Area and Porosity. London, NY: Academic Press.

[B12] Grela M. A. Colussi A. J. (1996). Kinetics of stochastic charge transfer and recombination events in semiconductor colloids. Relevance to photocatalysis efficiency. J. Phys. Chem. 100, 18214–18221. 10.1021/jp961936q

[B13] Jalajakumari N. Padmakumar N. Fujio M. Yoshinao O. Tatsuya O. (1999). Microstructure and phase transformation behavior of doped nanostructured titania. Mater. Res. Bull. 34, 1275–1290. 10.1016/S0025-5408(99)00113-0

[B14] Khalid N. R. Ahmed E. Hong Z. L. Zhang Y. W. Ullah M. Ahmed M. (2013). Graphene modified Nd/TiO_2_ photocatalyst for methyl orange degradation under visible light irradiation. Ceram. Int. 39, 3569–3575. 10.1016/j.ceramint.2012.10.183

[B15] Kočí K. Obalová L. Matějová L. Plachá D. Lacný Z. Jirkovský J. (2009). Effect of TiO_2_ particle siz on the photocatalytic reduction of CO_2_. Appl. Catal. B 89, 494–502. 10.1016/j.apcatb.2009.01.010

[B16] Kocí K. Troppová I. Edelmannová M. Starostka J. Matejová L. Lang J.. (2017). Photocatalytic decomposition of methanol over La/TiO_2_ materials. Environ. Sci. Pollut. Res. [Epub ahead of print]. 10.1007/s11356-017-0460-x 29043586

[B17] Lin H. Huang C. P. Li W. Ni C. Shah S. I. Tseng Y. H. (2006). Size dependency of nanocrystalline TiO_2_ on its optical property and photocatalytic reactivity exemplified by 2-chlorophenol. Appl. Catal. B 68, 1–11. 10.1016/j.apcatb.2006.07.018

[B18] Lu Z. Y. Jiang H. Q. Yan P. P. Li J. S. Wang Q. Y. (2011). Influences of Tm and N doping on surface properties and photoactivities of anatase-TiO_2_ nanoparticles. Adv. Mat. Res. 399–401, 519–526. 10.4028/www.scientific.net/AMR.399-401.519

[B19] Ma Y. Wang X. Jia Y. Chen X. Han H. Li C. (2014). Titanium dioxide-based nanomaterials for photocatalytic fuel generations. Chem. Rev. 114, 9987–10043. 10.1021/cr500008u 25098384

[B20] Maeda K. (2011). Photocatalytic water splitting using semiconductor particles: history and recent developments. J. Photochem. Photobiol. C 12, 237–268. 10.1016/j.jphotochemrev.2011.07.001

[B21] Maira A. J. Yeung K. L. Lee C. Y. Yue P. L. Chan C. K. (2000). Size effects in gas-phase photo-oxidation of trichloroethylene using nanometer-sized TiO_2_ catalysts. J. Catal. 192, 185–196. 10.1006/jcat.2000.2838

[B22] Matejova L. Brunatova T. Danis S. (2015). TiO2-CeO2 prepared by using pressurized and supercritical fluids: effect of processing parameters and cerium amount on (micro)structural and morphological properties. Res. Chem. Intermed. 41, 9243–9257. 10.1007/s11164-015-1990-9

[B23] Matějová L. Cajthaml T. Matej Z. Benada O. Kluson P. Solcova O. (2010). Super/subcritical fluid extractions for preparation of the crystalline titania. J. Supercrit. Fluids 52, 215–221. 10.1016/j.supflu.2009.12.008

[B24] Matějová L. Matěj Z. Fajgar R. Cajthaml T. Šolcová O. (2012). TiO2 powders synthesized by pressurized fluid extraction and supercritical drying: effect of water and methanol on structural properties and purity. Mater. Res. Bull. 47, 3573–3579. 10.1016/j.materresbull.2012.06.062

[B25] Meksi M. Turki A. Kochkar H. Bousselmi L. Guillard C. Berhault G. (2016). The role of lanthanum in the enhancement of photocatalytic properties of TiO_2_ nanomaterials obtained by calcination of hydrogenotitanate nanotubes. Appl. Catal. B 181, 651–660. 10.1016/j.apcatb.2015.08.037

[B26] Musić S. Gotic M. Ivanda S. Popovic A. Turkovic R. Trojko A. (1997). Chemical and microstructural properties of TiO_2_ synthesized by sol-gel procedure. Mater. Sci. Eng. B 47, 33–40.

[B27] Nie J. Mo Y. Zheng B. Yuan H. Xiao D. (2013). Electrochemical fabrication of lanthanum-doped TiO_2_ nanotube array electrode and investigation of its photoelectrochemical capability. Electrochim. Acta 90, 589–596. 10.1016/j.electacta.2012.12.049

[B28] Reli M. Ambrozova N. Sihor M. Matejova L. Capek L. Obalova L. (2015). Novel cerium doped titania catalysts for photocatalytic decomposition of ammonia. Appl. Catal. B Environ. 178, 108–116. 10.1016/j.apcatb.2014.10.021

[B29] Reli M. Huo P. Sihor M. Ambrozova N. Troppova I. Matejova L.. (2016). Novel TiO_2_/C_3_N_4_ photocatalysts for photocatalytic reduction of CO_2_ and for photocatalytic decomposition of N_2_O. J. Phys. Chem. A 120, 8564–8573. 10.1021/acs.jpca.6b07236 27701857

[B30] Reli M. Kobielusz M. Matějová L. Daniš S. Macyk W. Obalová L. (2017). TiO_2_ processed by pressurized hot solvents as a novel photocatalyst for photocatalytic reduction of carbon dioxide. Appl. Surf. Sci. 391, 282–287. 10.1016/j.apsusc.2016.06.061

[B31] Rengaraj S. Venkataraj S. Yeon J.-W. Kim Y. Li X. Z. Pang G. K. H. (2007). Preparation, characterization and application of Nd–TiO_2_ photocatalyst for the reduction of Cr(VI) under UV light illumination. Appl. Catal. B 77, 157–165. 10.1016/j.apcatb.2007.07.016

[B32] Schneider J. Matsuoka M. Takeuchi M. Zhang J. Horiuchi Y. Anpo M.. (2014). Understanding TiO2 photocatalysis: mechanisms and materials. Chem. Rev. 114, 9919–9986. 10.1021/cr5001892 25234429

[B33] Schneider P. (1995). Adsorption-isotherms of microporous mesoporous solids revisited. Appl. Catal. A 129, 157–165. 10.1016/0926-860X(95)00110-7

[B34] Siah W. R. Lintang H. O. Yuliati L. (2017). Role of lanthanum species in improving the photocatalytic activity of titanium dioxide. Catal. Sci. Technol. 7, 159–167. 10.1039/C6CY01991A

[B35] Troppová I. Lang J. Matějová L. (2017). Optimization of pressurized water and pressurized/supercritical methanol processing of Zr_0.1_Ti_0.9_O_n_ mixed oxide designed for mitigation of model dye Acid orange 7-polluted water Waste Forum 2, 64–76.

[B36] Wojcieszak D. Mazur M. Kurnatowska M. Kaczmarek D. Domaradzki J. Kepinski L. (2014). Influence of Nd-doping on photocatalytic properties of TiO_2_ nanoparticles and thin film coatings. Int. J. Photoenergy 2014:463034 10.1155/2014/463034

[B37] Xu X. Song W. (2016). Enhanced H_2_ production activity under solar irradiation over N-doped TiO_2_ prepared using pyridine as a precursor: a typical sample of N-doped TiO_2_ series. Mater. Technol. 32, 52–63. 10.1080/10667857.2015.1118587

[B38] Zhang Z. B. Wang C. C. Zakaria R. Ying J. Y. (1998). Role of particle size in nanocrystalline TiO_2_-based photocatalysts. J. Phys. Chem. B 102, 10871–10878. 10.1021/jp982948

